# Nanoparticle technologies in precision oncology and personalized vaccine development: Challenges and advances

**DOI:** 10.1016/j.ijpx.2025.100353

**Published:** 2025-07-05

**Authors:** Saber Imani, Samaneh Moradi, Tola Abdulsattar Faraj, Pejman Hassanpoor, Nazanin Musapour, Soran K. Najmaldin, Anno Hashm Abdulhamd, Aliasghar Tabatabaei Mohammadi, Chnar Husam Taha, Sargol Aminnezhad

**Affiliations:** a Shulan International Medical College, Zhejiang Shuren University, Hangzhou, Zhejiang, China; b Departments of Internal Medical, Shiraz University of Medical Sciences, Shiraz, Iran; c Department of Medical Analysis, Faculty of Applied Science, Tishk International University, Erbil, Iraq; d Department of Microbiology, Faculty of Basic Sciences, Rouzbahan Institute of Higher Education, Sari, Iran; e Clinical Research Development Center, 5th Azar Medical Center, Golestan University of Medical Sciences, Gorgan, Iran; f School of Medicine, Urmia University of Medical Sciences, Urmia, Iran; g Department of Molecular Genetics, Faculty of Biological Sciences, Tarbiat Modares University, Tehran, Iran

**Keywords:** Nanoparticles, Precision oncology, Drug delivery systems, Vaccine development, Immunotherapy

## Abstract

Nanoparticles (NPs) are changing the paradigm of precision oncology by providing means for targeted delivery, immune modulation, and personalized therapies for patients. To this end, drug delivery systems (DDS) have improved the precision in precision medicine and improved the design, delivery, and targeting of immune interventions through the use of NPs. This review aims to address the most clinically relevant NP platforms, including lipid (LNPs), polymeric (PNPs), metal-based (MNPs), ceramic (CNPs), carbon-based (CBNs), aptamer conjugated (ANPs), and quantum dots (QDs), and reviewed as potential therapeutic and diagnostic applications and their utility in oncology. Further, we will touch on next-generation systems, including hybrid NPs (HNPs), stimulus-response NPs (SRNPs), and artificial-intelligence (AI) directed NPs (AI-NPs) that are programmable and adaptive with precision-engineered capabilities for cancer vaccinations and immunotherapy. We will discuss how NPs function as a DDS and how these systems facilitate controlled antigen release, better delivery to antigen-presenting cells, and the delivery of neoantigen-based immunotherapies. The ability of NPs to support cell-based therapies, including CAR-T cells, and help overcome multi-drug resistant (MDR) is also explored. Although obstacles remain regarding the development of scalable, safe, and regulatory approved therapies, the ongoing progress in the field of nanomedicine suggests new strategies enabling the delivery of efficient personalized anticancer therapies with clinical benefits for cancer patients.

## Graphical abstract

**Figure:**
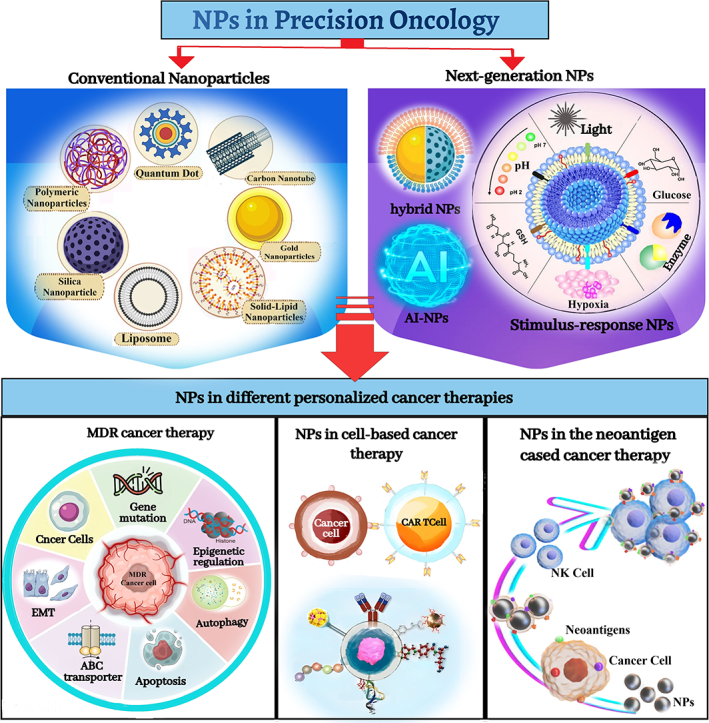

## 1 Introduction

Nanoparticles (NPs) are disrupting precision oncology by creating new avenues to targeted drug delivery, diagnostic imaging, and immunotherapy (Hristova-Panusheva et al., 2024). NPs are typically 1 to 100 nm in diameter, have the principal features of being highly surface area to volume ratios, tunable surface chemistry, and the unique ability to cross biological barriers including the blood-brain barrier (Cheng et al., 2012; Davis and Mensah, 2017; Kango et al., 2013). These properties allow preferential delivery of therapeutic agents to tumor sites, enhance treatment efficacy, and reduce toxicity, which are key components of individualized cancer treatment (Chehelgerdi et al., 2023a; Luobin et al., 2024). There are many types of NPs being developed and studied for anticancer drug delivery or detection of tumor-specific biomarkers such as lipid (LNPs), polymeric (PNPs), metallic (MNPs), ceramic (CNPs), carbon-based (CBNs), aptamer-conjugated (ANPs), and quantum dots (QDs). There are also antibodies on nanoparticle platforms such as Au-NPs and IO-NPs used to bind to certain receptors on the cell surface of cancer cells like HER2 and EGFR, which then carry treatment options including chemotherapy or CRISPR therapy to the cancer cells (Afzal et al., 2022). For example, Doxil® is a drug formulated in polyethylene glycol (PEG)-conjugated liposomal formulations, and will capitalize on the enhanced permeability and retention effect to deliver the chemotherapeutic agent doxorubicin to cancerous tissue (Chen et al., 2016). *NPs, particularly LNPs, are essential for receptor-mediated uptake of RNA-based therapies such as mRNA and siRNA* inside tumor cells in such a way that can modify or stimulate gene expression. The platform can be engineered to co-deliver immunomodulators and immune checkpoint inhibitors (ICIs) alongside tumor-associated antigens (TAAs), synergistically enhancing antitumor immune responses (Tripathi et al., 2023). NPs also offer cancer vaccines with neoantigens and adjuvants to a variety of tumor types, especially combinations of those to combat multidrug resistance (MDR), or cold tumors (Chenthamara et al., 2019; Imani et al., 2024b; Liu et al., 2020). NPs are being evaluated and integrated into co-delivery systems for antigens and immune adjuvants. For example, the combination of Au-NPs and PLGA-based vectors will increase the stability of the antigens and promote the better cell internalization and uptake within cells, and it therefore increases the potential effectiveness of the vaccine (Yu et al., 2023). Au-NPs are an array of promising tools for cancer immunotherapy owing to its unique immunomodulatory properties. Recent studies proved that AuNPs could trigger powerful T-cell-mediated immune responses in dendritic cells, this ability increased, when AuNPs were coupled with tumor antigens or adjuvants (Toraskar et al., 2022). Moreover, AuNP-based systems enhanced cytokine secretion by almost 10 times to levels of IL-6 and TNF-α to trigger innate immune activation (Sun et al., 2021). In addition, their use in cancer vaccine platforms have demonstrated enhanced antigen presentation and cancer specific immunity targeting indicating that AuNPs can be a strong immunotherapy platform of next generation (Trabbic et al., 2021).

Because CNPs are thermally stable and biocompatible, they are highly suitable for dual imaging and treatment. CBNs enhance electrical and mechanical properties for controlled loading and release of drugs. Additionally, ANPs offer possible targeting and diagnostic capabilities. Overall, systems with these properties can offer amazing possibilities to personalize cancer treatments in a very individualized way. In this review, we explore the most advanced NP systems clinically NH, LNP, PNP, MNP, CNP, CBN, and ANP types, and their applications in cancer diagnosis and treatment. We give focus to also next-generation. NP types, hybrid NP (HNP), stimuli-responsive NP (SRNP), and artificial intelligence-optimized NP (AI-NP) allowing solutions for multifunction. HNP, is the amalgamation of organic and inorganic materials for simultaneous therapy and imaging; SRNP, relies on tumor microenvironment (TME) responses to release payload; and AI-NP are computationally built on targeting (Mba and Nweze, 2021). Clinical translation is hindered due to safety features like immune recognition, systemic toxicity, and the need for repeatable scalability in NP synthesis. Many theranostic NP, HNPs, are exciting by utilizing real-time image modification allowing real-imaging therapy monitoring (Lozano et al., 2023; Wang et al., 2022b). As nanotechnology advances, particularly with mRNA delivery and gene-editing systems, nanoformulations (sized a few nanometers to hundreds of nanometers) will likely help researchers move past established barriers and eventually implement safe, effective, and patient-specific oncologic therapies.

## 2 Types of NPs

### 2.1 LNPs

**Fig. 1:**
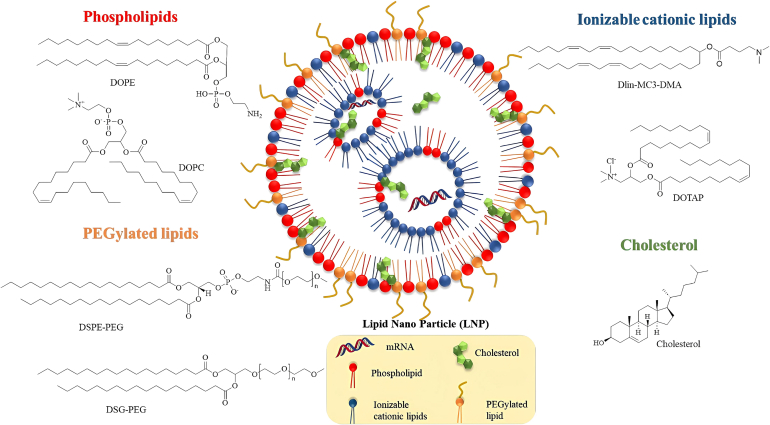
**Chemical structures of compounds used in lipid nanoparticle (LNP) formulations.** The LNP formulations include phospholipids such as DOPE (1,2-dioleoyl-sn-glycero-3-phosphoethanolamine) and DOPC (1,2-dioleoyl-sn-glycero-3-phosphocholine), which contribute to membrane stability and fluidity. Cholesterol is another critical component, enhancing structural integrity and reducing permeability. Ionizable cationic lipids, including DOTAP (1,2-dioleoyl-3-trimethylammonium-propane) and Dlin-MC3-DMA (1,2-dilinoleyloxy-3-dimethylaminopropane), play a key role in endosomal escape and cargo delivery. Additionally, PEGylated lipids, such as DSPE-PEG (1,2-distearoyl-sn-glycero-3-phosphoethanolamine-N-[methoxy(polyethylene glycol)]) and DSG-PEG (1,2-distearoyl-sn-glycero-3-phosphoethanolamine-N-[succinyl(polyethylene glycol)]), are incorporated to improve colloidal stability and reduce immune recognition, ensuring efficient systemic delivery of therapeutic agents.

LNPs provide a successful drug delivery platform by improving drug stability, solubility and bioavailability while enhancing targeted delivery and controlled release (Shah et al., 2020). These nanocarriers address important pHarmaceutical issues including enzymatic degradation, systemic toxicity, and poor absorption rates. When it comes to distributing cytotoxic agents, antibiotics and nucleic acid agents, liposomes are best (Wei et al., 2024). LNPs are important drug delivery systems in mRNA therapies due to their ability to protect RNA from degradation, uptake by cells and assist in cytosolic release of the drug payload. They are composed of phospholipids and cholesterol (which form a stable lipid bilayer), ionizable lipids (which bind and encapsulate the drug), and PEG-conjugated lipids (for improving systemic circulation and decreasing immune detection)(Fig. 1) (Cheng and Lee, 2016).

LNPs mimic cell membranes, enable direct intracellular delivery (Mehta et al., 2023). LNPs mimic cell membranes, enable direct intracellular delivery (Mehta et al., 2023). When LNPs are placed in acidic environments within the endosome, their stability decreases and their cargo is released into the cytosol, which is important for their therapeutic effects. Key properties such as drug-loading capacity, encapsulation efficiency, and particle stability under physiological conditions are critical for effectiveness of LNP-based DDS (Kulkarni et al., 2019; Samad et al., 2007; Shah et al., 2020; Xu et al., 2022). Table 1 summarizes approved liposomal drugs and their clinical applications. These formulations provide precision targeting, increased bioavailability, and better safety profiles. For instance, Doxil®, a PEGylated liposomal doxorubicin (DOX), was approved in 1995 and, because of its longer circulation time and decreased cardiotoxicity, has become a mainstay in the treatment of Kaposi's sarcoma, multiple myeloma, and ovarian cancer (Chen et al., 2024; Santhanakrishnan et al., 2024).

**Table 1:** Approved LNPs formulations and their applications.

Product	API	Year	Clinical application	Mechanism	Key benefits	References
Doxil®	DOX	1995	OC, MM, KS	PEGylated liposomes for prolonged T½	Reduced cardiotoxicity, stable drug	Santhanakrishnan et al. (2024)
DaunoXome®	DNR	1996	HIV-KS	Unilamellar vesicles for targeted delivery	Lower systemic toxicity	Rosenthal et al. (2002)
DepoCyt®	Ara-C	1999	LM	Sustained-release in CSF	Prolonged action, CNS targeting	Domínguez et al. (2005)
Marqibo®	VCR	2012	ALL	Sphingomyelin-based liposomes	Higher tolerability, fewer off-target effects	Shah et al. (2016)
Onivyde®	IRN	2015	MPC	Liposomal PK improvement	Enhanced efficacy, less GI toxicity	Liu et al. (2018)
Vyxeos®	Ara-C + DNR	2017	AML	Synergistic fixed-ratio liposomes	Improved survival in AML	Alfayez et al. (2020)
Mepact®	MFT	2009	Osteosarcoma	Macrophage-targeted delivery	Boosts immune response	Jiménez-López et al. (2021)
Lipusu®	PTX	2006	BC, NSCLC, OC	Solvent-free liposomal delivery	Safer, reduced hypersensitivity	Regenold et al. (2022)

Abbreviations list: API, active pharmaceutical ingredient; OC, ovarian cancer; MM, multiple myeloma; KS, Kaposi's sarcoma; LM, lymphomatous meningitis; CNS, central nervous system; ALL, acute lymphoblastic leukemia; AML, acute myeloid leukemia; MPC, metastatic pancreatic cancer; BC, breast cancer; NSCLC, non-small cell lung cancer; NTM, non-tuberculous mycobacteria; PK, pharmacokinetics; GI, gastrointestinal; T½, half-life; DOX, doxorubicin; DNR, daunorubicin; Ara—C, cytarabine; VCR, vincristine; IRN, irinotecan; MFT, mifamurtide; PTX, paclitaxel.

Approved in 2015, Onivyde®, a liposomal formulation of irinotecan is now indicated for the treatment of metastatic pancreatic cancer and has improved drug retention and decreased gastrointestinal toxicity Other approved liposomal formulations include DaunoXome® for HIV associated Kaposi's sarcoma, DepoCyt® for lymphomatous meningitis, Marqibo® (sphingomyelin-based liposomal vincristine for acute lymphoblastic leukemia), and Vyxeos® (liposomal fixed-ratio combination cytarabine and daunorubicin formulation for acute myeloid leukemia) (Alfayez et al., 2020; Rosenthal et al., 2002). Mepact®, a formulation of mifamurtide, enhances immune activation against osteosarcoma and was approved by the FDA in 2009 for osteosarcoma patients with no metastasis. Lipusu®, a paclitaxel (PTX) liposome formulation was solvent free and provides a significantly safer PTX for breast, lung and ovarian cancer patients (Jiménez-López et al., 2021).

In addition to these examples, immunoliposomes designed with enhanced targeting using ligand targeting of tumor specific markers, like EGFR-DOX (Lee et al., 2023). For example, during clinical trials for TNBC, anti-EGFR immunoliposomes in which DOX is coupled to the IgG component are being evaluated (Mamot et al., 2023). New generation liposomes that have been developed, such as Talidox® are in Phase I clinical trials intended to assess safety and efficacy (Mc Laughlin et al., 2024). Clinical trials are also underway for liposomal topotecan, which is a hydrophilic anticancer drug delivery formulation, in Phase I clinical trials in patients with advanced solid tumors (Matulonis et al., 2022). Thermo DOX, thermosensitive liposomes which required test methods have completed Phase III but had worse outcomes in patients with liver cancer and other solid tumors (Regenold et al., 2022). For care, in a mouse model of leiomyoma, for example liposomal simvastatin was trialed with reduced tumor volumes and Ki67 expression, however, were not statistically significant. The study illustrated how LNPs may be able to improve pharmacokinetics of poorly bioavailable drugs, such as simvastatin (El Sabeh et al., 2022). Innovative approaches have evolved in LNP formulation by developing flexible liposomes using edge activators, which entail using Tween 80 or cholate to promote penetration through the skin. These formulations can demonstrate higher transdermal efficacy compared to rigid liposomes. Targeted delivery with peptide-conjugated liposomes represents yet another innovation. For example, pegylated liposomal DOX carrying AR13 peptides localizes to MUC1 receptors on colon cancer cells, and demonstrated both an elevated uptake and a prolonged live for pegylated liposomal DOX attached to AR13 peptides as compared to the standard Doxil® (Biabangard et al., 2023). Curcumin-loaded pluronic F127-liposomes (CUR-LIP-F127) demonstrated significantly increased wound healing, and enhanced the stimulation of keratinocyte migration, while enhancing the Nrf2/Keap1 signalling (Biabangard et al., 2023)(Zhou et al., 2023). Finally, an example of the use of liposome combination chemotherapy, was illustrated using co-encapsulation of salinomycin and gemcitabine for colorectal cancer (CRC). The two drugs illustrated synergistic cytotoxicity and continuously released the drugs through a 72-h period, which demonstrates an application of the design of experiments strategy to optimize physicochemical properties and therapeutic effects (Tefas et al., 2023).

### 2.2 PNPs

**Fig. 2:**
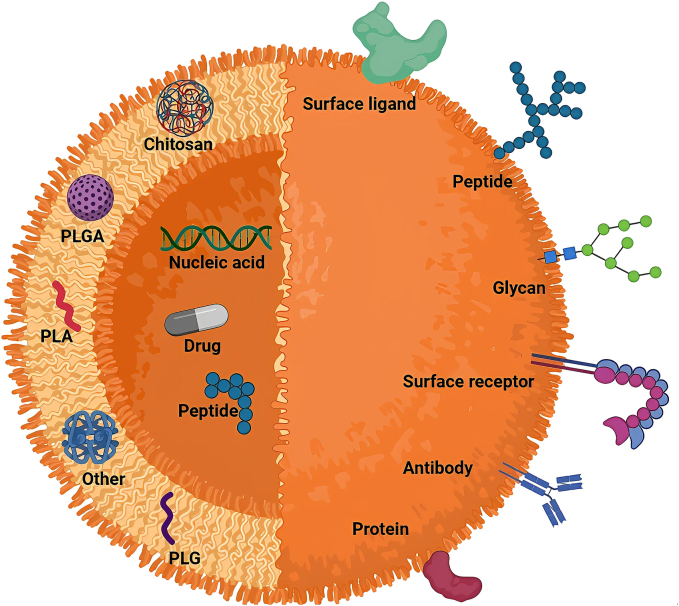
**Structure of a PNP.** This figure depicts the structural design of polymeric nanoparticles (PNPs), which consist of a polymer-based matrix that encapsulates active pharmaceutical ingredients (APIs). The core of the nanoparticle is where the drug is loaded, while the surrounding polymeric shell is engineered to regulate the drug's release over time. The polymer matrix serves as a stabilizer, ensuring that the encapsulated drug is protected from premature degradation. This controlled release ensures that the drug is gradually delivered to the target tissue, thus minimizing side effects by reducing exposure to healthy tissues. Additionally, the outer polymer shell can be modified with specific ligands or antibodies, enabling the nanoparticles to target specific receptors on diseased cells (e.g., cancer cells). This enhances the therapeutic efficacy of the drug and ensures that it is delivered precisely where it is needed, making PNPs ideal for applications in cancer treatment, chronic disease management, and targeted infection therapies.

PNPs are derived from either natural or synthetic substances and are vital in DDS because they significantly allow the tailored effect delivery of useful agents (Akram et al., 2023). Biodegradable polymers, such as PLA, PLGA, and PCL are particularly special examples; they have biodegradability, so they are less toxic since they degrade to products that resolve with the body and are more compatible as the body has biodegradability tolerance. Natural polymers, such as chitosan, alginate, gelatin, and albumin, provide flexible platforms as they have good drug release profiles (Mehrotra et al., 2023). Recent advances have demonstrated the use of chitosan-based PNPs for DOX encapsulation. The characteristics and physiochemical properties of these NPs were confirmed by X-ray diffraction and UV–Vis spectroscopy which confirmed that the chitosan NPs were amorphous and possessed a high thermal stability. Another useful characteristic of these systems was the pH-responsive nature that allowed for drug release in the the low pH TME was feasible (Akram et al., 2023). PLA-based NPs have also been used for the co-delivery of navitoclax (NAV) and decitabine (DCB) creating a synergistic effect in both AML and breast cancer models. As detailed by Mehrotra et al., the dual loaded NAV/DCB NPs were taken up by >95 % of cells within 2 h, localized to the mitochondria, exhibited hemocompatibility, and inhibited tumor growth substantially in THP-1 and WEHI-3B leukemia cells (Fig. 2). (Mehrotra et al., 2023). PNPs have showed promising effectiveness in medical treatments of Leishmania infections apart from their established role in cancer therapy. Scientists developed RIF-PTM-PNPs which they incorporated within Carbopol gel for topical usage. The topical application of NPs in gel base allowed them to dwell on the skin which led to prolonged drug release and better drug absorption. The drug delivery system enhanced drug activity against Leishmania infection by increasing macrophage uptake since this cellular process is essential for effective Leishmania treatment. The PNPs can be optimized through this method for topical drug delivery which guarantees prolonged and exact treatment at infection sites (Khan et al., 2023a). In a similar way, Pandey et al. examined PLA NPs loaded with tamoxifen(TMX) with greater Efficacy of TNBC treatment and lesser immunological side effects and hepatic and renal toxicity (Pandey et al., 2015). As per an additional study, β-sitosterol was loaded in PLGA and PEG-b-PLA NPs and was confirmed to have cytotoxic activity against MCF-7 and MDA-MB-231 breast cancer cells through MTT assay and flow cytometry (Andima et al., 2018). (Andima et al., 2018; Yin et al., 2022). The incorporation of amphiphilic PCL-based polymers that can simultaneously deliver PTX and DOX have been successful by Yin and colleagues. The linking of PTX and encapsulated DOX into liposomes achieved loading at 11.6 % and 12.4 %, respectively. The engineered NPs released the most drug at a pH resembling the TME (Yin et al., 2022). Narisepalli et al. have developed asiaticoside (AST)-loaded PNPs that showed increases in many parameters when compared with free AST in terms of wound closure rates and wound healing quality and improved fibroblast migration works, collagen synthesis, and tissue regeneration to augment diabetic wound healing in rats (Narisepalli et al., 2023). Another promising use exists with the chemotherapeutic drug GEM with PNPs produced with chitosan and polyharmonies. Formulations were successful in increasing GEM loading efficiencies and cellular uptake. In addition, tumor-specific delivery occurred with surface modifications of EGFR variant III (EGFRvIII) ligands that are tumor-specific in OC to increase the amount of drug accumulated intratumorally (Bhattacharya et al., 2022). Overall, PNPs have widespread biomedical applications including tissue regeneration, control of infectious diseases, and cancer treatment. The versatility of PNPs in their design by modifying polymer composition and surface architecture allows targeted delivery, controlled drug release, and reduced systemic toxicity. All these advancements are examples of the innovative role that PNPs play in contemporary treatment modalities, as noted in Fig. 2 and described in Table 1.

### 2.3 MNPs

They are very useful for targeted drug delivery because of their unique optical properties and surface modifications. Modified noble metal nanoparticles (MNPs), including silver (Ag), gold (Au), palladium (Pd), platinum (Pt), titanium (Ti), zinc (Zn), and copper (Cu), have enhanced optical properties and biocompatibility. Surface modifications can be made through covalent, hydrogen, and electrostatic interactions, which facilitates targeted attachment of biomolecules that improve tissue specificity. Many drugs and therapeutic compounds, like chemotherapy, nucleic acids, peptides, and antibodies, are carried very efficiently by MNPs (Chandrakala et al., 2022; Zhuo et al., 2024).

Surface modifications can be made to MNPs that contain targeting ligands, polymers, or bio-molecules that improve specificity, stability, and therapeutic efficacy. Recent studies demonstrate that MNPs can improve drug delivery efficacy and decrease side effects. Oliveira et al. developed manganese ferrite NPs coated with lipid that had PTX for the targeted treatment of malignant melanoma. By adding a magnetic field to weaken off-target effects, these NPs were able to release PTX within tumors. Their IC50 was 142,500 times lower than free PTX. (Oliveira et al., 2023). MNPs are also extremely important and promising to imaging, diagnostics and combination therapies; thus, they are a flexible modality in both current and modern medicine. Similarly fabricated GBP-Ag-NPs (GBP loaded Ag-NPs) and demonstrated greater anticancer effects on A549 LC cells with 50 % cell death at GBP concentration of 150 Μm (Yaseen et al., 2023). Hernandes et al. examined DOX loaded iron oxide NPs (IrO-NPs-DOX) in breast cancer (MCF-7 cells), and find that they had enhanced drug uptake, DNA damage and inhibited cell migration with magnetism (Hernandes et al., 2023). Tyagi et al. synthesized superparamagnetic iron oxide NPs (BSA-SPIONs-TMX) by green methods conjugating TMX, which were non-toxic in preclinical studies and reduced MCF-7 and T47D BC cell growth (Tyagi et al., 2023). The research conducted by Mukherjee et al., found that PEGylated platinum (Pt-NPs-DOX) showed better tumor suppressive and apoptosis inducing abilities in cancer cells compared to conventional forms of chemotherapy (Mukherjee et al., 2020; Patel et al., 2020). Kim et al., developed TiO-NPs-DOX that were conjugated with phenylboronic acid (PBA) and also created an ultrasound activated platform which showed strong anticancer behavior against MCF-7 and MDA-MB-231 BCE cells (Kim et al., 2020). Research conducted by George et al. showed that loading curcumin, quercetin, and naringenin using L-histidine-conjugated chitosan with ZnO-NPs demonstrated an HNPs system that had a drug loading capacity greater than 90 % and also showed between 15 and 30 times greater cytotoxic effect on A431 skin cancer cells (George et al., 2020). (George et al., 2020). Cu-NPs (Copper Nanoparticles) are also emerging in use to treat cancer. With the goal of co-delivering disulfiram and Cu, Wu et al. created a PEGylated hollow mesoporous Si-NPs (DSF@PEG/Cu-HMSNs) to further prevent tumor growth, which was capable of generating very cytotoxic CuET complexes and ROS within tumors (Wu et al., 2019). Rozalen et al. also developed AgNPs coupled with methotrexate that decreased tumor growth in CRC and lung cancer models, while also reducing systemic toxicity to zebrafish (Rozalen et al., 2020). Progress in the research of MNPs (Metallic Nanoparticles) highlights their importance in the crusade against cancer in modern times. MNPs provide support for cancer treatment via increasing drug solubility, targeting medicines to the required location, and decreasing harmful side effects (Mukherjee et al., 2020; Patel et al., 2020). With advancements in MNP engineering, surface functionalization and combination therapies there is expected to be an expansion in the variations MNPs will be clinically beneficial (Shanthi et al., 2015; Tyagi et al., 2023).

### 2.4 CNPs

CNPs have emerged as very promising platforms for cancer therapy based on their chemical stability, biocompatibility and tunable drug delivery properties (Echalar et al., 2023). The majority of these NPs consist of metal oxides, carbides, phosphates, and carbonates with silicon, calcium, and titanium representing typical examples. CNPs can offer temperature stability, chemical inertness, controls on porosity, and effective drug encapsulation and subsequent release in an pH independent manner. MSNs are investigated the most because they have large pore sizes in unique to tune the pore size enough to allow them to load multiple therapeutic agents (Iturrioz-Rodríguez et al., 2019; Moreno-Vega et al., 2012; Sutthavas et al., 2022). Functionalization of MSN with ligands or polymers also increases targeting specificity. MSNs with curcumin (Cur-MSNs) have previously shown selective cytotoxicity against HN5 head and neck cancer cells by lowering Bcl-2 expression, increasing the Bax/Bcl-2 ratio, and inducing ROS-mediated apoptosis (Sharifi et al., 2022). In the same vein, Chang et al. defined DOX@MSN-PEI-AA (DMPA) for delivering DOX via the Sigma receptor into TNBC cells after conjugating polyethyleneimine (PEI) and anisamide ligands. Anisamide enhanced receptor-mediated uptake while PEI prompted endosomal escape to limit premature drug releases and increase therapeutic efficacy Another versatile class of CNPs includes calcium phosphate NPs (CaP-NPs). They have excellent binding affinity for chemotherapeutics such as cisplatin (CDDP), DOX, and PTX along with pH sensitivity and biocompatibility. Chen et al. constructed hollow amino-functionalized CaP-NPs (CaPO-NH2) using a PAMAM dendrimer template and subsequently loaded them with 5-fluorouracil (Chang et al., 2022). These NPs accomplished controlled intracellular release and reached normal cells with acceptable biocompatibility and was able to kill PSN1 cells through ROS production and calcium ion overload (Chen et al., 2022; Qiu et al., 2022). The importance of NPs design in enhancing cancer treatment is noted with the shape-dependant efficiencies of this system (Shen and Ma, 2022). In their study, the hexagonal prism shaped core-shell mesoporous Si-NPs (CSMS) loaded with cabazitaxel had considerably better drug uptake and release from prostate cancer cells than their spherical shape, confirming that design and efficiency are highly important in this system (Mohanan et al., 2024). These systems provide stability, membrane penetration ability and avoid enzymatic degradation to optimize mRNA cytoplasmic release (Li et al., 2023; Lin et al., 2024a). At the acidic TME or endosomal sites, (specially CaP-NPs) responsive disassembly promotes the intracellular release of TAA-encoding mRNA, which achieves long-lasting antitumor immunity by inducing cytotoxic T lymphocyte responses and activating DCs (Bose et al., 2022). The specificity of antigen-presenting cells is further enhanced by targeting at least some surface ligands on the NPs to reduce off-target effects. All of these CNP-based mRNA vaccine systems provide “customized” immune activation with a long-term therapeutic benefit, which is important for personalized cancer immunotherapy (Ghemrawi et al., 2024).

### 2.5 QDs

QDs are emerging in personalized oncology due to their unique optical characteristics of tunable fluorescence, high photostability, and feasibility for functionalization and targeted delivery. Typically, QDs are far brighter and fluorescent longer than ordinary organic dyes, thus they are perfect candidates for early detection, imaging, visualizing, and tracking cancer progression to determine efficacy of treatment. An enormous strength of QDs is their surface adaptability they can be functionalized with ligands, peptides, or antibodies for targeted recognition of tumors. This ability has led to incorporation of diagnosis with treatment in a single system as a nanotheranostic platform(Khan et al., 2023b). Haider et al. devised a multifunctional nanotheranostic system by employing GO-QDs especially conjugated to GILGFVFTL peptide, which binds with PLAC-1 an over-expressed tumor-associated antigen in CRC cells (Haider et al., 2023). Not only did it completely spare PLAC-1 negative cells, but the QD-peptide conjugate (QD-P) showed selective internalization into PLAC-1+ HT-29 and HCT-116. This also confirmed targeting efficacy of the peptide while demonstrating PLAZ-1 specificity by having an antagonizing effect by down-regulating the PLAZ-1 expression. Decreasing their invasiveness and potential for cancer cell metastasis. QD-P was able to selectively internalize into PLAC-1+ HT-29 and HCT-116 while avoiding PLAC-1- due to the quantitative and spatially selective internalization and this demonstrated both the specificity of the peptide as a targeting moiety and anti-cancer capacity of the peptide through downregulation of PLAC-1 expression and invasiveness and potential to metastasize. Another study focusing on the use of QDs in personalized cancer therapy was done by Liu et al. who created silicon-based quantum dots (Si-QDs) and functionalized them with chlorin e6 (Ce6) and PBA for combined photodynamic therapy and photothermal therapy. Si-QDs@Ce6/PBA NPs increased targeting and cellular uptake. On stimulation with light, the system generated reactive oxygen species (ROS) for inducing apoptosis in tumor cells, while PBA increased NPs deposition in tumors(Liu et al., 2023). Also, QDs might also be used for mRNA-based cancer vaccines. QDs allow long-term, real-time, high-resolution imaging and monitoring of the delivery, biodistribution, and cellular internalization of mRNA vaccines, given the targeting efficiency provided by QDs confinement effect and stable fluorescence (Gorle et al., 2023). QDs also have a tunable emission spectrum and this provides the ability for multiplex imaging, which is not achievable with traditional NPs. This useful feature allows us to track and monitor multiple tumor sites or components of a tumor at once during the course of immunotherapy.(Liu et al., 2021). Overall the multifunctional capabilities of QDs- including selective tumor delivery, long-term tracking, enhanced imaging, and protection of sensitive genetic material- represent a powerful and highly capable vehicle for personalized vaccines and therapeutics for cancer.

### 2.6 CBNs

**Fig. 3:**
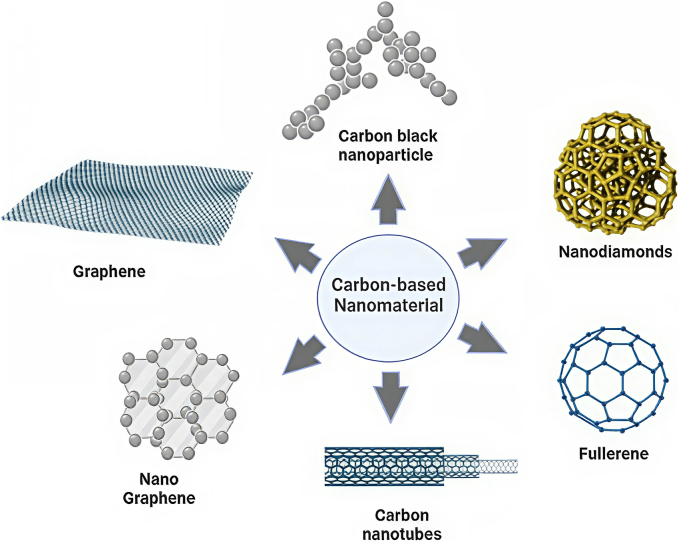
**Carbon-based nanomaterials (CBNs) in cancer therapy and personalized vaccine development.** This figure illustrates the potential of carbon-based nanomaterials (CBNs), such as carbon nanotubes (CNTs), graphene oxide (GO), and carbon quantum dots (CQDs), as drug delivery systems (DDS) in personalized cancer therapy. CBNs are functionalized to carry chemotherapeutic agents like doxorubicin (DOX) or curcumin, ensuring targeted delivery to tumor sites with high precision. Their pH-sensitive properties enable controlled drug release in the acidic tumor microenvironment, enhancing therapeutic efficacy while minimizing systemic toxicity. Additionally, CBNs can be modified with targeting ligands for selective binding to cancer cells, improving therapeutic outcomes in personalized medicine. This figure demonstrates how CBNs can be tailored for the development of personalized mRNA cancer vaccines and as nanocarriers for drug delivery, offering a promising strategy to increase the specificity and effectiveness of cancer treatments.

CBNs are nanoscale structures comprised mostly of carbon atoms and typically between 1 and 100 nm in size. As in Fig. 3, CBNs exhibit extraordinary physicochemical properties - including very high surface area, electrical conductivity, mechanical strength, and widely varying chemical and structural morphologies. The CBN family consists of 6 main classes of NPs; graphene, nanodiamonds, carbon QDs (CQDs), fullerenes, carbon nanotubes, and carbon nanohorns (Danafar et al., 2022). These materials have a range of functional advantages that create opportunities for application in drug delivery, bioimaging, tissue engineering, electronics, and cancer therapies. There have been several promising developments in DDS that involve CBN-mediated delivery of chemotherapeutics and mRNA vaccines, based on both the intrinsic properties of CBNs, and specifically their surfaces that are compatible with ligands, the field of personalized oncology has derived significant benefit (Díez-Pascual, 2021). Microwave-synthesized, pH-sensitive CBNs have been used to deliver DOX to cancer cells, with drug release enhanced within the acidic TME, which improved treatment efficacy. In vitro studies demonstrated that CBNs-DOX can induce apoptosis and provide an effective approach for targeted delivery in the MDA-MB-231 cells(Dechsri et al., 2023). CBNs have also been used for the controlled release of natural anticancer agents. For example, curcumin-loaded CBNs with bovine serum albumin (BSA) (chemical name) showed pH-sensitive release and reduced breast cancer cell growth, which demonstrated the possibilities for safer and effective personalized cancer medicines (Danafar et al., 2022). CBNs have also been used in photothermal therapy (PTT), particularly CuO-functionalized CBNs. PTT uses a hybrid nanomaterial to convert light to heat to induce targeted destruction of cancer cells. CuO greatly increased the PTT performance and provides a non-invasive approach for targeted ablation of tumors (Jiang et al., 2020). CBNs' targeted delivery of metformin can eliminate hepatocellular carcinogenic stem cells, decrease tumor burden, and increase the efficacy of the drug. This demonstrates that CBNs can be used for highly aggressive cancers and cancers that are resistant to treatment (Sun et al., 2023). Ultrasound-assisted labeling of CBNs has been established for the detection of metastatic lymph nodes in TNBC patients before neoadjuvant chemotherapy. This showed the capability to accurately assess the metastasis status of lymph nodes, which provides a means to accurately direct staging surgery for better surgical outcomes and lower recurrences (Lin et al., 2022). The targeting capacity of CBNs have been further enhanced by functionalizing GO and CNTs with tumor-specific ligands. CQDs also hold intrinsic fluorescence providing in vivo tracking ability of mRNA vaccination delivery and therapeutics distribution(Tang et al., 2022). With targetable moieties like peptides or antibodies, CBNs have the potential to limit off-target toxicity because of their accurate bioavailability at tumor sites, which is a critical determination for personalized oncology. CBNs are able to deliver custom mRNA vaccines containing TAAs into the TME and elicit significant immune responses. The surface chemistry of the CBNs will protect RNA from degradation, increase delivery efficiencies, and increase the therapeutic index(Gao et al., 2024). The CBNs have exciting and multifunctional potential as they pertain to the co-delivery of both chemotherapeutics and mRNA cancer vaccines (Fig. 3). The targeting capacity and pH-sensitivity of CBNs have the potential to improve rigor and efficacy in the surgical therapeutics pathway while decreasing overall systemic side-effects.

### 2.7 ANPs

Aptamers are small, single-stranded DNA or RNA molecules that adopt a distinct three-dimensional structure to bind with high specificity and affinity to target molecules (e.g. proteins, peptides, as well as whole cells). Their molecular recognition capabilities represent a very powerful new class of therapeutics, diagnostics and, drug deliverable agent. In the context of oncology, aptamers represent a low weight, low immunogenic, specific and targeted delivery system with substantial potential for numerous applications in both cancer therapeutics and cancer care for the public health sector (Lakhin et al., 2013). The functional application of aptamers for malignant targeting, depends first on the use of tumor-specific biomarkers for binding. For instance, the antibody like aptamer to epithelial cell adhesion molecule (EpCAM) applomer binds specifically to EpCAM which is a biomarker predominantly over-expressed in epithelial derived neoplasms such as cancer of the breast, colon and, ovary. Combining the ANP drug delivery platform with an aptamer will be tumor specific while sparing adjacent healthy tissue (Szymanowski et al., 2023). The sgc8 aptamer binds to protein tyrosine kinase-7, which is over-expressed in leukemia, CRC, and breast cancer, and provides a targeted technologies for potential of therapeutic targeting (Sicco et al., 2023). ANP DDS is expanding rapidly. Aptamers can be attached to many other NPs, like liposomes, MNPs, etc. that may result in even more specific delivery (Aminnezhad et al., 2024b). For instance, anti-EGFR aptamers attached to Au-NPs may target EGFR expressing tumor cells in lung, head, neck, and colon cancers. This cleared system improved therapeutic uptake and minimized nonspecific interactions and off-target toxicity (Song et al., 2012). In addition, Au-NPs can utilize plasmon resonant light to free drug unmasking, which will help determine the specificity and cytotoxic efficacy of a treatment (Song et al., 2012; Wei et al., 2022). ANPs are also formulated as gene therapy vectors. That is, delivering siRNAs or miRNAs through sgc8 aptamers to cancer cells silences oncogenes that drive tumor growth and survival. Personalized medicine strategies will implement aptamer based gene therapies to optimize therapeutic specificity. The CD30 aptamer targeted the chemotherapeutics delivery to CD30+ lymphoma cells where it showed improved efficacy while reducing systemic toxicity (Luo et al., 2019). DDSs that utilize liposomes decorated with aptamers possess enormous potential. For example, MUC1 aptamer targets the MUC1 glycoprotein, which is overexpressed on breast cancer cells. The MUC1 aptamer was incorporated in a liposome system containing CDDP and was shown to have better targeting of tumors and substantially reduced toxicity (Yu et al., 2011). A similar, but slightly different system that also utilized QDs with MUC1 aptamers and DOX (QD-MUC1-DOX) describes an aptamer-directed delivery system. The pH-sensitive QD-MUC1-DOX conjugate released DOX from the payload only after the QD-MUC1-DOX had been internalized into MUC1-overexpressing ovarian cancer cells to avoid any MDR, which ultimately improved the efficacy of the therapeutics (Savla et al., 2011). Protein-based NPs that have been functionalized with aptamers allow for targeted therapeutics. For example, MS2 bacteriophage capsids functionalized with the photosensitizer, when evaluating AS1411, were found to advance photodynamic therapy for non-small cell lung cancer. Following light activation, aptamer-directed molecules selectively kill cancer cells while sparing adjacent normal tissues (Wang et al., 2022a; Zhou and Rossi, 2014). In terms of vaccine delivery, aptamers can increase performance for mRNA and DNA vaccines. In mRNA vaccines, targeted delivery using an aptamer improves the cell uptake of both DCs and tumor cells that activate and present antigens (Zhou and Rossi, 2014). Ultimately, ANPs provide an extremely flexible and focused platform to produce personalized cancer immunotherapy, which targets more safely and effectively (Xiang et al., 2015).

### 2.8 Next-generation NPs

Next-generation NPs systems are taking precision cancer immunotherapy to the next level by overcoming some of the significant limitations in cancer therapy, including tumor heterogeneity, immune evasion and personalizing treatment regimens. These exciting new engineering, target responsive, and adaptive formulation designs (Chenthamara et al., 2019). Description of the unique characteristics of LNPs also facilitates inviting additional new tools such as gene editing technology, and cellular therapies such as CAR T-cell therapy (Aminnezhad et al., 2024a; Mahaling and Katti, 2016). The combination of the new technologies is disrupting the therapeutic realm, whence they are providing patient-centered, multi-functional treatments, and personalized with respect to their individual treatment needs and tumor biology (Chou et al., 2023; Mitchell et al., 2021). The combined advancements are revolutionizing the therapeutic space and thus providing treatment options that are personalized, multi-functional, and tailored toward the individual needs of patients and the biology of their tumors (Bhange and Telange, 2025; Samathoti et al., 2025). This next section will review how each NP class is distinct and pre-clinical implementations based on selected food classes, with a special focus on mRNA-based cancer vaccines and immunotherapy (Chou et al., 2023; Mitchell et al., 2021).

#### 2.8.1 HNPs

Table 2 provides information indicating that HNSs are multifunctional platforms comprising both organic (e.g., lipids, polymers) and inorganic (e.g., silica, iron oxide, QDs) materials (Kim, 2025). For example, in stage III models of triple-negative breast cancer (TNBC), lipid–mesoporous silica NPs are used to co-deliver mRNA encoding tumor-specific neoantigens and CpG oligo nucleotides. The lipid shell enhances endosomal escape, while the silica core enhances structural stability and payload capacity. This combination allows for enhanced cross-presentation on MHC-I, leading to strong CD8^+^ T-cell responses and regression of the tumor (Chen et al., 2009; Darvishi et al., 2017). For glioblastoma multiforme (GBM), lipid-coated iron oxide NPs (LC-IO-NPs) co-deliver the PD-L1 siRNA and tumor lysate antigens. Therefore, this platform modulates immune checkpoint regulation and activates antigen-specific T-cells. Because the silica core is iron oxide, it is possible to use LC-IO-NPs for MRI and theranostic imaging (Hsieh et al., 2022). Finally, in HER2^+^ breast cancer, QDs–lipid composites (QD-LNPs) have been reported to facilitate both siRNA delivery (targeting HER2) and photothermal therapy. Exposure to near-infrared light inhibits HER2 signalling and induces cytotoxicity in the tumor. Importantly, QD-LNPs had ∼30 % greater accumulation in the tumor when compared to the non-hybrid technology (Bouras et al., 2015; Di Filippo et al., 2021).

**Table 2:** Emerging NPs platforms and innovations in cancer therapy.

NP type	Key mechanism	Clinical advantages	Emerging innovations	Standout findings	References
LNPs	mRNA encapsulation/protection	FDA-approved; high transfection efficiency	Stimuli-responsive (pH/temp); AI-optimized lipid composition	85 % tumor reduction in melanoma; superior to viral vectors in safety	Imani et al. (2024b); Wei et al. (2024)
PNPs	Controlled drug release	Biodegradable (PLGA); co-delivery potential	PLGA-metal hybrids; ML-guided polymer design	94 % tumor suppression in AML with navitoclax + decitabine	Huang et al. (2024); Mehrotra et al. (2023)
MNPs	Magnetic/optical properties	Hyperthermia synergy; imaging capability	Au-PEG thermo-responsive systems; AI-based surface modifications	142,500× IC₅₀ reduction in melanoma with PTX-loaded MNPs	Oliveira et al. (2023); Wu et al. (2019)
CNPs	Curcumin delivery	Apoptosis induction; mitochondrial pathway targeting	Redox-sensitive surface-engineered NPs	3.43× increase in Bax/Bcl-2 ratio in HN5 and A549 cells	Sharifi et al. (2022); Shen and Ma (2022)
QDs	PLAC1-targeting	Anti-metastatic; improved cytotoxicity and imaging	QD-bioconjugates for real-time tumor tracking	Reduced metastasis and cytotoxicity in HT-29 and HCT-116 CRC models	Haider et al. (2023)
CBNs	pH-sensitive DOX release	CSC targeting; tumor-selective accumulation	Graphene‑carbon dot hybrids for dual therapy	Enhanced DOX release in MDA-MB-231 and HCC models	Dechsri et al. (2023); Sun et al. (2023)
ANPs	EGFR targeting (aptamer-AuNPs)	Specific tumor targeting; reduced off-target toxicity	Aptamer-nanogold platforms for RNA delivery	High selectivity, low toxicity in EGFR^+^ lung and colon cancer models	Song et al. (2012); Savla et al. (2011)
HNSs	Organic–inorganic synergy	Multifunctional therapy; enhanced delivery and targeting	LNP–MSN hybrids; QD-lipid composites	30 % higher tumor accumulation vs. single-component systems	Lin et al. (2024a); Shirzad et al. (2025)
SRNPs	TME-triggered activation	Precision drug release; reduced off-target effects	pH-sensitive micelles; redox-activated dendrimers	5× increase in drug bioavailability at tumor sites	Alshawwa et al. (2022); Chen et al. (2022)
AI-NPs	AI-guided design optimization	Accelerated development; personalized formulations	Neural network-PK/PD prediction; generative AI for materials	40 % improvement in tumor targeting accuracy	Bhange and Telange (2025); Samathoti et al. (2025)

Abbreviations list: NPs, nanoparticles; LNPs, lipid nanoparticles; PNPs, polymeric nanoparticles; MNPs, metallic nanoparticles; CNPs, ceramic nanoparticles; QDs, quantum dots; CBNs, carbon-based nanoparticles; ANPs, aptamer-functionalized nanoparticles; HNSs, hybrid nanosystems; SRNPs, stimuli-responsive nanoparticles; AI-NPs, artificial intelligence-designed nanoparticles; PLGA, poly(lactic-*co*-glycolic acid); ML, machine learning; Au-PEG, gold-polyethylene glycol; IC₅₀, half maximal inhibitory concentration; PTX, paclitaxel; DOX, doxorubicin; EGFR, epidermal growth factor receptor; AuNPs, gold nanoparticles; TME, tumor microenvironment; MSN, mesoporous silica nanoparticle; PK/PD, pharmacokinetics/pharmacodynamics; CRC, colorectal cancer; HCC, hepatocellular carcinoma; CSC, cancer stem cells.

#### 2.8.2 SRNPs

SRNPs are designed to release therapeutic cargo to their target site, namely, the tumor, upon specific stimuli that occur from tumor cells, for example, changes in pH, redox potential, hypoxia, or to the proteolytic enzymes (Table 2) (Alshawwa et al., 2022; Chang et al., 2021; Ma and Jiang, 2018; Teo et al., 2017; Zhang et al., 2022). When SRNPs are in the acidic tumor microenvironment (TME), the micelles disassociate due to the change in pEG conformation and release their cargo in the cytosol, eliciting the cGAS–STING pathway, DCs maturation, and secretions of type I interferon (Boudier et al., 2009). In platinum-resistant OC, predominantly using glutathione-responsive dendrimers (GSH-RDs) for selective devliery of PTX and neoantigen mRNA to cancer cells overproducing GSH (Criscuolo et al., 2022). The GSH-RDs cause Bax/Bcl-2-mediated mitochondrial apoptosis and produce a cytotoxic T-cell response that overcomes chemoresistance (Eckstein, 2011). In stage II-III CRC, MMP-9 cleavable PNPs (MMP9-PNPs) target KRASG12D mutations by delivering mRNA vaccines specifically to MMP-9 rich invasive tumor regions (Hong et al., 2025). This targeted delivery improves antigen presentation and clonal expansion of effector CD8+ T-cells, thus reducing in vivo tumor volume.

#### 2.8.3 AI-NPs

AI-NPs, described in Table 2, employ computer-based modelling including machine learning (ML), deep neural networks (DNNs), and generative algorithms (GA) to optimize the properties of NPs, predict in vivo pharmacokinetic/pharmacodynamics (PK/PD) parameters, and personalize formulations using tumor-specific omics profiles (Kumar et al., 2022). The use of these development cycles which rely on data-driven platforms leads to inference and enables true personalized immunotherapy. As an example, in cutaneous melanoma, machine learning-based tools were utilized to design combinations of LNPs based on the HLA alleles and tumor mutational burden of each patient (Imani et al., 2024a). These AI-designed formulations of lipoplexes (AI-LNPs) retain mRNA stability and enhance systemic delivery to promote durable CD4^+^/CD8^+^ T-cell response in resistant models and those that do not respond to immune checkpoint blockade. In cases of pancreatic ductal adenocarcinoma (PDAC), which has an identifiable immunosuppressive stroma, AI-designed PEGylated lipid-polymer hybrids (AI- LPHs) can co-deliver mutant KRAS mRNA and STING agonists that activate IRF3/NF-κB signalling cascades and neoantigen spreading to promote T-cell inclusion and limit metastatic spread in KPC mouse models of PDAC (Xu et al., 2025). For EGFR-mutated lung cancer, ML-optimized NPs (ML-NPs) were used to deliver both EGFR inhibitors and mRNA vaccines to neoantigens (Imani et al., 2025). This mirrors a two-prong strategy: inhibition of growth pathways coupled with stimulation of adaptive immunity through prime and boost vaccination, providing prolonged tumor regression only to resistance (Arandhara et al., 2025; Crintea et al., 2023).

## 3 NPs in different personalized cancer therapies

**Fig. 4:**
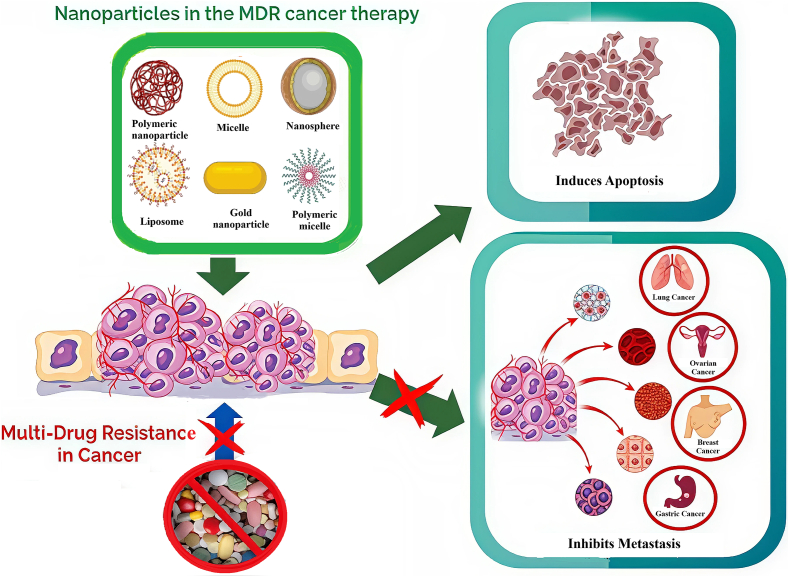
**Nanoparticles in multi-drug resistant (MDR) cancer therapy.** This graphic highlights the role of nanoparticles in overcoming multi-drug resistance (MDR) in cancer. MDR poses a major obstacle in effective cancer treatment, where cancer cells develop resistance mechanisms, including efflux pumps, anti-apoptotic signalling, and enhanced DNA repair. Nanoparticles, such as liposomes, dendrimers, polymeric micelles, and quantum dots, offer a targeted solution. They are engineered to bypass resistance mechanisms by delivering chemotherapeutics directly into cancer cells, ensuring sustained intracellular drug concentrations. The enhanced permeability and retention (EPR) effect facilitates the preferential accumulation of nanoparticles in tumors due to their leaky vasculature. Surface modifications on nanoparticles, such as antibodies or ligands, enable active targeting of MDR cells. Additionally, nanoparticles can co-deliver therapeutic agents with MDR modulators like siRNA, further sensitizing cancer cells to chemotherapy. This leads to apoptosis induction in resistant tumors and reduces metastasis to distant organs, such as the lungs, ovaries, stomach, and breasts. The figure illustrates how nanoparticles are a promising tool for addressing MDR and improving cancer therapy outcomes.

NPs-based DDSs are revolutionizing cancer therapy toward the ultimate goal of personalized medicine with high precision and efficiency in therapeutic delivery. In comparison to conventional drug delivery systems, NPs are able to site selectively possess high efficiency and controlled release of therapeutic cargos at tumor sites with minimal off-target toxicity in non-cancerous tissues (Fig. 4). Furthermore, their Circulation and Distribution or Interaction in a Biological Environment is also mediated by the design characteristics like Size-Composition-Surface charge of NPs themselves (Chenthamara et al., 2019). Current applications of NPs in cancer are MDR, gene editing and increasing efficacy of cellular therapies (e.g., CAR T cells) [302]. The future is challenging With improved technology, we can formulate cancer treatment strategy based on the molecular and genetic profile of each patient (Mahaling and Katti, 2016). Following are expanded opportunities in the paragraphs below.

### 3.1 NPs in the MDR cancer therapy

MDR is an important obstacle to cancer therapy as it allows malignant cells to evade the cytotoxic effects of different therapies. MDR accounts for treatment failure and uncontrolled tumor growth. Traditional chemotherapeutic agents primarily use single-agent treatment regimens. Malignant cells develop resistance mechanisms, through increased drug efflux, enzymatic degradation or alteration of target site therefore diminishing the patient's therapeutic response. NP systems could be designed to contain combinations of drugs that induce efficacy through synergy; this could help overwrite MDR in cancer cells and eventually overcome treatment resistance (Shi et al., 2022). As shown in Fig. 4, NPs can potentially confront MDR by targeting efflux mechanisms, can selectively target cancer cells which are drug resistant, and can co-deliver sensitizing agents such as siRNA. Their effective tumor-specific delivery also allows NPs enhanced selective accumulation to the tumor site by enhanced permeability retention effect, overcoming heterogeneity. Indeed heterogeneous tumors (breast cancer, lung cancer and ovarian cancer) are often observed to have MDR phenotypes. The most prominent mechanism of resistance is drug efflux via P-glycoprotein. NPs systems can also co-deliver anticancer agents with inhibitors of MDR protein, restoring drug sensitivity (Wang et al., 2017). In contrast, this may have a significant clinical impact on patients who do not have a response to standard therapies, in part because they have either inherited or acquired the resistance(Wang and Huang, 2020). Liposomes- spherical vesicles with a phospholipid bilayer- are one of the most widely studied NP-based DDS, while allowing for both hydrophilic and hydrophobic drugs. The presence of PEGylated liposomes (e.g., oxaliplatin-loaded PEGylated LNPs) further extends the circulation time while decreasing immune clearance of the liposomes, decreasing not only the clearance rate of the administered dosage, which increases drug bioavailability, but may also decrease side effects (Çağdaş et al., 2014). Co-delivery systems are equally relevant in the context of MDR. Modified liposomes with hyaluronic acid encapsulating hesperetin and CDDP have demonstrated a stronger anticancer effect in triple-negative breast cancer. The HA-functionalization of the liposomes will allow for preferential uptake by tumor cells with a high CD44 expression, which is expressed in breast cancer. In addition, pH and enzyme-responsive NP systems co-encapsulating disulfiram and DOX induced powerful anti-metastatic effects in TNBC metastasis (Çağdaş et al., 2014; Wang et al., 2023). These developed NPs platforms enhance drug accumulation and tumor targeting, while also enabling personalized intervention via tumor-specific biology. With this, NP designed mediated strategies provide a novel approach in overcoming MDR and contributing to improving patient survival.

### 3.2 NPs in cell-based cancer therapy

**Fig. 5:**
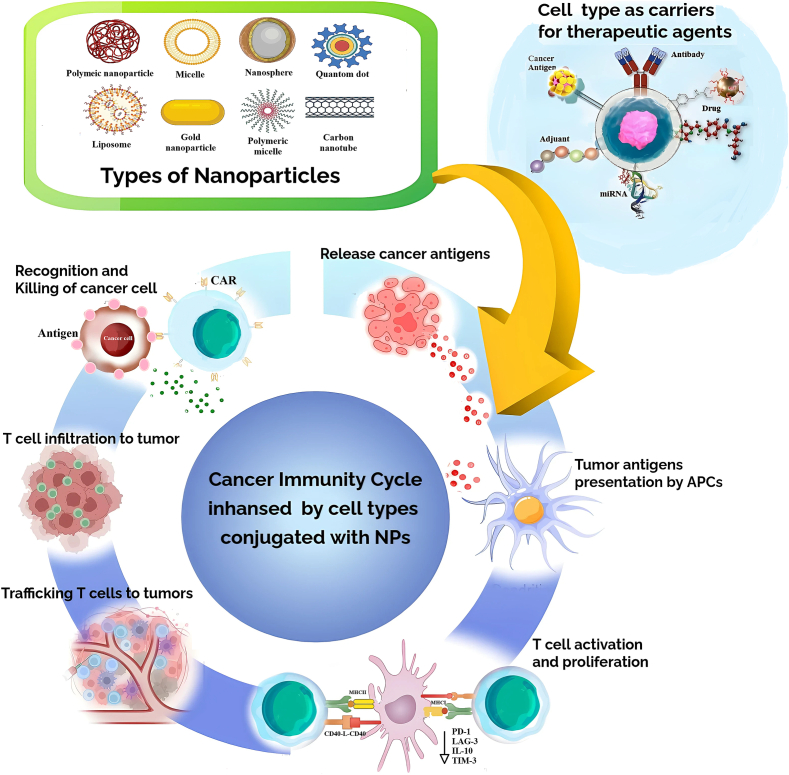
**Cancer immunity cycle enhanced by nanoparticles in persolizned cancer therapy.** A schemtics demonstrates how different types of nanoparticles, such as liposomes, polymeric nanoparticles, dendrimers, and metallic nanoparticles, serve as carriers for therapeutic agents to enhance the cancer immunity cycle. The cycle begins with the release of tumor antigens, which are then processed and presented by antigen-presenting cells (APCs) to activate T cells. The activated T cells proliferate and are trafficked to the tumor site, where they infiltrate the tumor microenvironment and recognize cancer cells. This process ultimately leads to the elimination of cancer cells. By integrating nanoparticles with immune cells, the immune response is significantly amplified, improving the efficiency of tumor targeting and destruction.

The combination of cell-based NPs with NPs represent a combined steps forward in the prospects of a cancer treatment modality. Delivery can now be improved with respect to the specifications of its therapeutic agents, the stabilization of therapeutic agents, and controlled dosage, duration, and success was the efficacy. Engineered cells can carry NPs containing chemotherapies, immunomodulators, or RNA-based therapies for spatial and temporal release for the TME (Fan et al., 2023; Parveen et al., 2023). As we show in Fig. 5, this represents a considerable advancement for anti-cancer immunotherapy that optimizes many parts - phase - of the cancer-immunity (e.g., antigen release, antigen presentation, T cell activation, T cell infiltration, and T cell-mediated cytotoxicity). NPs can also optimize the therapeutic capacity of small extracellular vesicles (sEVs), which are naturally secreted, lipid bilayer-bound carriers derived from T cells, DCs, or mesenchymal stem cells, that exhibit inherent biocompatibility and immune modulating capabilities to evade immune response mechanisms making them ideally suited to deliver drugs. sEVs can efficiently and safely release their RNA, protein, or small molecule payloads to target cells by bio-activity mediated by membrane fusion. The combination of sEVs with engineered NPs, such as liposomes or MSNs, increases delivery accuracy. For example, IL-2 modified sEVs containing miRNAs against Bcl-2 and VEGF stimulated immune responses, tumor suppression and anticancer actions. The generative ability of sEVs to NPs was based on their biocompatibility and immune evasion to function as natural vectors to deliver drugs or small molecules. sEVs, being composed of RNA molecules, proteins and/or small molecules, also have a system to deliver their carrying cargo from the forming host cell to the target cell by using the host cell's membrane system (Boca et al., 2020; Hang et al., 2023; Moghassemi et al., 2024) The choice of NPs type is based on tumor characteristics, as well as the drug or therapeutic properties. For example, liposomes which consist of a phospholipid bilayer which is similar to the cell membrane, can be used to encapsulate both hydrophobic and hydrophilic drugs and ideally with sEVs (Wang et al., 2023). MSNs with high surface area and tunable porosity can be used as carriers for siRNAs, chemotherapeutics, or immune modulators. These studies are a step in the right direction, as MSNs can overcome immune evasion in models of melanoma and pancreatic cancer (Hang et al., 2023). PNPs such as PLGA can offer periodic drug release profiles along with dual-action therapeutic uses in gene therapy and chemotherapy in numerous cancers, including glioblastoma and ovarian cancer (Mansouri et al., 2021). Synthesizing sEVs with engineered NPs, including liposomes or MSNs, results in a more accurate delivery. For example, IL-2-modified sEVs loaded with miRNAs directed against Bcl-2 and VEGF were able to improve immune responses and tumor suppression. This type of concerted sEV and NP engineering would shortly take advantage of sEVs biocompatibility and innate immune evasion as naturally made vectors to deliver drugs or small molecules. sEVs have been shown to carry RNA molecules, protein, and/or small molecules and possessed a mechanism to uniquely deliver that cargo from the forming host cell into the target cell by membrane fusion (Yashchenok et al., 2023). These systems allow for the controlled delivery of anti-cancer compounds to targeted tumors expressing (MDR) or immune-related traits (e.g., sEVs containing mRNA or miRNAs targeting Bcl-2) (Lin et al., 2024b). Nanoparticle (NPs) delivery into CAR T-cell therapy is novel, especially for the treatment of solid tumors that often reject these therapies due to physical and immune barriers. NPs improve CAR T-cell trafficking, enhance intratumoral retention, and improve activation. In a murine model of hepatocellular carcinoma, CAR T-cells loaded with mesoporous silica nanoparticles (MSNs) and activated by photothermal therapy (PTT) improved targeting of tumor mass and local cytotoxicity. Likewise, T-cell membrane-coated NPs (N3-TINPs) worked analogous to native immune cells by improving biodistribution and localization resulting from a combination of cellular and NP distribution, which enhance the treatment option to incorporate a dual immune and heat-based strategy. As solid tumors tend to present a physical barrier and immunological resistance to traditional CAR T-cell therapies, the potential addition of NPs into CAR T-cell therapies gives rise to new possibilities for addressing solid tumors. NPs can support CAR T-cells in routine trafficking to and accumulation in the intertumoral site of action with activation of NPs. The reported use of MSNs, loaded into CAR T-cells, and activated via PTT has been shown to improve tumor targeting and local cellular cytotoxicity in hepatocellular carcinoma models (Fan et al., 2023; Paramanantham et al., 2022). Additionally, engineered NPs that have T-cell membranes are more closely related to the biodistribution and approved tumoral localization of native immune cells, allowing for a safe dual delivery of immune and heat-activated therapies (Han et al., 2019). In order to further overcome the immunosuppressive TME, NPs have also been used to directly deliver immune-activating cytokines (ex: IL-12, IL-15) and immune checkpoint inhibitors (ICIs)(ex: anti-PD-1 and anti-CTLA-4) to tumor sites. This localized delivery sustains CAR T-cell function and augments their persistency in the hostile TME (Oliveira et al., 2024). As such, the ability of NPs to deliver individual agents for solid tumors and the genetic heterogeneity in solid tumors – such as RNAs either encoding proteins or mRNA molecules, and small drugs that may target mutations found only in the patient - may represent a new development in precision oncology. Though NP-facilitated cell-based therapies have a lot of potential, limitations remain particularly regarding immune evasion, long term stability, and safety of nano systems. Innovative designs and functionalization of NPs will be required to fine-tune their therapeutic properties in Clinical Oncology (Han et al., 2019; Nawaz et al., 2020).

### 3.3 NPs in the neoantigen-cased cancer therapy

**Fig. 6:**
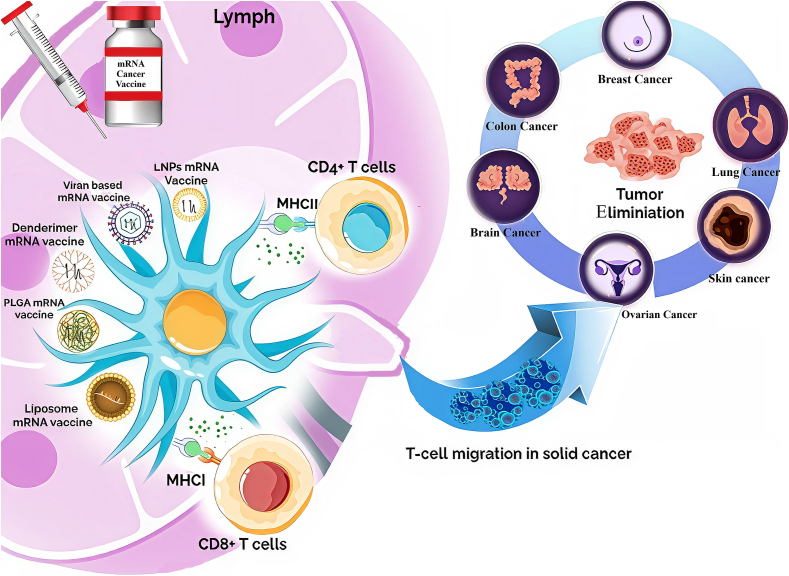
**Nanoparticles-based mRNA cancer vaccines and antigen personalization.** This figure outlines the mechanism of action of mRNA vaccines delivered through nanoparticles, such as lipid nanoparticles (LNPs), dendrimers, PLGA, and liposomes, in tumor antigen presentation and vaccination efficacy. The nanoparticles carry mRNA encoding tumor-specific antigens, which are delivered to dendritic cells in the lymphatic system. These cells process the mRNA and present the resulting antigens via MHC molecules, activating CD4+ helper T cells and CD8+ cytotoxic T cells. Once activated, the T cells migrate to the tumor site, infiltrate the solid tumor, and attack cancer cells. The figure highlights the potential of this approach for targeting and eliminating various tumor types, making nanoparticle-based mRNA vaccines a promising advancement in cancer immunotherapy.

The recent addition of mRNA-based vaccines against tumor-specific neoantigens are highly innovative and redefined the scope of cancer immunotherapies. With the ability to individualize treatment regimens, mRNA vaccines present a unique opportunity in cancer immunotherapy. An important limitation of mRNA therapy is in general its stability and rapid degradation in the circulation, which proves to be challenging for effective delivery. Encapsulating mRNA is critical to its delivery method and there are many NP delivery possibilities, while LNPs are among the most well-studied and promising drug delivery systems imparting protection to mRNA and deliver to its target location. LNPs are the most promising NP delivery platform based on biocompatibility, delivery capabilities and protecting characteristics to allow mRNA to withstand enzymatic degradation (Qu et al., 2024). In Fig. 6, the pathways of LNPs and other types of NPs such as PNPs, and PLGA-based NPs, are shown, the pathways mRNA vaccines take to DCs, while the NP transport biodistribution occurs to many tissues fit for immune response. NPs not only stabilize mRNA through circulation, they also enhance DC uptake of mRNA through endocytosis, and a DC uses mRNA; it transcribes the mRNA in cytoplasm to generate tumor-specific neoantigens. Neoantigens are then processed and presented on major histocompatibility complex (MHC) molecules in two ways, MHC I and MHC II; when engaged on MHC I this directly activates CD8^+^ cytotoxic T lymphocytes, and when engaged on MHC II that directly activates CD4^+^ helper T cell responses in immune response coordination through cytokine secretion. CD4^+^ T cells coordinate the immune response through cytokine release and CD8^+^ T cells directly lyse tumor cells by destroying tumor cells that Neomaxis recognize as distinct to neoantigens (Imani et al., 2024b; Kiousi et al., 2023). LNP formulations have continued to be the base platform for mRNA cancer vaccine development, showing both a promising delivery method and efficient translational potential. Clinical studies have shown positive outcomes in melanoma and lung cancer about LNP-formulated mRNA vaccines using personalized neoadjuvants on an individual level based on their tumor mutational profile (Imani et al., 2024b). In particular, surface functionalization allows tumor-targeting ligands to engage with the mRNA and improves specificity and efficacy (Jacob et al., 2024; Zhou et al., 2022). These vaccines produce strong cytotoxic T cell responses and can have improved therapeutic results when used alongside ICIs, such as PD-1/PD-L1 antibodies. ICIs will mitigate the immune suppression mediated by tumors and improve the response to mRNA vaccines (Kiousi et al., 2023). Their biodegradability and biocompatible attributes make them suitable for clinical applications, and their slow-release characteristics increase the duration of antigen presentation and immune activation (Huang et al., 2024).

Dendrimers, which have a highly branched nanoscale structure, offer yet another versatile platform. LNP-encapsulated mRNA vaccines are already being investigated for a number of cancers, including breast cancer and glioblastoma. For example, HER2/neu-targeted mRNA vaccines delivered in LNPs also demonstrated promising immunogenicity and tumor reduction in pre-clinical models of breast cancer HER2^+^ (Tapescu et al., 2024). Overall, NP platforms such as LNPs, PNPs, and dendrimers are playing a central role in the progress of personalized mRNA-based cancer therapies. These types of technology allow the protection and targeted delivery of tumor-specific neoantigens, ultimately increasing immune modulation and leading to tumor eradication (Parvin et al., 2024).

## 4 Challenges and future directions

Even if NP-based precision oncology and personalized vaccines hold great potential, major challenges need to be addressed before their clinical implementation can be realized (Gavas et al., 2021). One of the main challenges is the selective delivery of therapeutic agents to tumor cells without affecting neighboring healthy tissues (Rasool et al., 2022). Traditional treatment modalities, such as chemotherapy and radiotherapy, ultimately have non-selective cytotoxic effects that can limit their effectiveness. While NPs have the capability for both passive and active targeting, effective and consistent delivery can be considerably compromised by the heterogeneity of the TME and abnormality of the vessels (Chehelgerdi et al., 2023b; Yao et al., 2020). Considerations for ensuring prolonged circulation time, tumor accumulation, controlled drug release, and clearance that does not have toxicity to normal healthy cells still requires novelty and improvement to be realized (Yao et al., 2020). In addition, NP biocompatibility and hronic toxicity still need to be assessed (Abbasi et al., 2023). NPs need biodegradable materials that have minimal toxicity to prevent persisting in the body without negative consequences after medical intervention. The safe clinical application of NPs demands full comprehension about their biological interactions during extended usage periods (Nkanga, 2023).There are technical and economic hurdles to the translation of NP fabrication from the laboratory to industrial scale (Muthu and Wilson, 2012). Processes such as solvent evaporative/solvent exchange and emulsion polymerization cannot simply be scaled up in a reproducible way (Herdiana et al., 2022) Processes such as solvent evaporative/solvent exchange and emulsion polymerization cannot simply be scaled up in a reproducible way (Herdiana et al., 2022). Given that regulatory guidelines are still in formation, and coordinated approvals from the FDA and EMA remain underdeveloped (Joseph et al., 2023; Rodríguez et al., 2022). Future progress in nanoparticle technology will rely on overcoming key challenges, such as integrating diagnostic and therapeutic functions, ensuring precise delivery, and effectively stimulating immune responses for more personalized and effective cancer treatment.--- (Sun et al., 2024). (Priyanka et al., 2023). (Li et al., 2022). Specifically, the precise delivery of immunotherapeutics may resolve a number of issues related to systemic immune activation (Aljabali et al., 2024). Major considerations are the design of NPs that are bio-safe and biodegradable (biodegradable polymers). Natural biodegradable polymers are gaining traction in the literature, especially PLA and CS due to their compatibility with biocompatible and benign degradation profiles (Hussain et al., 2024). Surface modifications are also crucial for controlling NP size, shape and charge to promote penetration into tumors and limit off-target effects (Mitchell et al., 2021). Fortunately, NPs bring innovative ways of treating central nervous system tumors by closely passing through the BBB, which is generally impossible for standard therapies (Janjua et al., 2023). Modulating therapy with chemotherapy, immunotherapy, and gene therapy can provide a more effective and comprehensive treatment paradigm by utilizing NPs. NPs also fight resistance mechanisms and provide increased efficacy of standard therapies (Gorbet and Ranjan, 2020). In conclusion, NPs offer new applications for actual smart NPs, hybrid NPs, and NPs with artificial intelligence capabilities that potentially represent new insight for the treatment of CNS tumors. Smart NPs react to changes in tumor micro-environments and can use variations like pH gradients and redox gradients to manage release (Alshawwa et al., 2022), and HNPs allow for synergy, or simultaneous treatment option such as chemo-photothermal therapy (Shirzad et al., 2025). In addition, AI NPs can also use variation of physicochemical parameters and predict biological interactions (Bhange and Telange, 2025; Samathoti et al., 2025). Continuous modifications to achieve these technologies with coordinated efforts at the international level to facilitate regulatory harmonization and scaling the manufacturing process of NPs so that NPs could eventually normalize at the clinical level is common highest level to achieve in oncology standard care. If we are to succeed at achieving many of these developments, toe realization of NP therapy can transform personalized cancer care treatment that can bring about greater safety consideration with better targetability and efficiency (Đorđević et al., 2022).

## 5 Conclusion

NPs can act as a basis for the development of cancer therapies and vaccines through delivery technologies that can enhance an effect while having lower systemic toxicity. Our work describes the promise of mRNA-loaded LNPs and PNPs carriers as neoantigen vaccines, which could provide more targeted immune activation and allow treatment that is more subsequently personalized, aiding patients fighting neoplasms. However, the long-term safety, manufacturability, and regulatory issues that are still present in the use of nanomedicine continue to be hurdles; as different fields of science come together it will allow the full potential of nanomedicine clinical avenues to develop.

## CRediT authorship contribution statement

**Saber Imani:** Writing – review & editing, Writing – original draft, Visualization, Methodology, Formal analysis, Conceptualization. **Samaneh Moradi:** Writing – review & editing, Methodology, Investigation, Conceptualization. **Tola Abdulsattar Faraj:** Writing – review & editing, Methodology, Investigation. **Pejman Hassanpoor:** Writing – review & editing, Supervision, Methodology, Conceptualization. **Nazanin Musapour:** Writing – review & editing, Methodology, Conceptualization. **Soran K. Najmaldin:** Writing – review & editing, Methodology. **Anno Hashm Abdulhamd:** Writing – review & editing. **Aliasghar Tabatabaei Mohammadi:** Writing – review & editing, Writing – original draft, Visualization, Methodology, Investigation, Formal analysis, Conceptualization. **Chnar Husam Taha:** Writing – review & editing. **Sargol Aminnezhad:** Writing – review & editing, Writing – original draft, Visualization, Methodology, Formal analysis, Conceptualization.

## Ethics approval and consent to participate

Not applicable.

## Funding

This work was supported by the Talent Scientific Research Project of 10.13039/501100005356Zhejiang Shuren University [grant numbers KXJ1723104, 2021].

## Declaration of competing interest

The authors have no competing interests.

## Data Availability

Data will be made available on request.
